# High-throughput 454 resequencing for allele discovery and recombination mapping in *Plasmodium falciparum*

**DOI:** 10.1186/1471-2164-12-116

**Published:** 2011-02-17

**Authors:** Upeka Samarakoon, Allison Regier, Asako Tan, Brian A Desany, Brendan Collins, John C Tan, Scott J Emrich, Michael T Ferdig

**Affiliations:** 1 Department of Biological Sciences, Eck Institute for Global Health, University of Notre Dame, Notre Dame, IN 46556, USA; 2 Department of Computer Science and Engineering, University of Notre Dame, Notre Dame, IN 46556, USA; 3 454 Life Sciences, a Roche company, 15 Commercial Street, Branford, CT 06405, USA

## Abstract

**Background:**

Knowledge of the origins, distribution, and inheritance of variation in the malaria parasite (*Plasmodium falciparum*) genome is crucial for understanding its evolution; however the 81% (A+T) genome poses challenges to high-throughput sequencing technologies. We explore the viability of the Roche 454 Genome Sequencer FLX (GS FLX) high throughput sequencing technology for both whole genome sequencing and fine-resolution characterization of genetic exchange in malaria parasites.

**Results:**

We present a scheme to survey recombination in the haploid stage genomes of two sibling parasite clones, using whole genome pyrosequencing that includes a sliding window approach to predict recombination breakpoints. Whole genome shotgun (WGS) sequencing generated approximately 2 million reads, with an average read length of approximately 300 bp. *De novo *assembly using a combination of WGS and 3 kb paired end libraries resulted in contigs ≤ 34 kb. More than 8,000 of the 24,599 SNP markers identified between parents were genotyped in the progeny, resulting in a marker density of approximately 1 marker/3.3 kb and allowing for the detection of previously unrecognized crossovers (COs) and many non crossover (NCO) gene conversions throughout the genome.

**Conclusions:**

By sequencing the 23 Mb genomes of two haploid progeny clones derived from a genetic cross at more than 30× coverage, we captured high resolution information on COs, NCOs and genetic variation within the progeny genomes. This study is the first to resequence progeny clones to examine fine structure of COs and NCOs in malaria parasites.

## Background

Advances in genotyping technology led to an explosion of studies to identify genes of interest using classical genetic approaches [1]. Such studies facilitate the discovery of genetic factors related to disease, drug resistance and environmental response. Different approaches evolved rapidly with improvements in sequencing technology. Additional advances in molecular biology techniques have greatly increased the speed and throughput of discovery and analysis. For example, microarray-based marker discovery has been applied to model organisms such as yeast [2], *Arabidopsis *[3,4], rice [5-7], and non model organisms including the human malaria parasite *Plasmodium falciparum *[8-11]; however, this platform can be susceptible to poor hybridization efficiency of low complexity regions and difficulties in reproducibility. Such problems are magnified in organisms with high nucleotide bias, particularly the extreme case of *P. falciparum - *80.6% (A + T) composition [12], resulting in limitations in genome-wide coverage and cost effectiveness.

Alternatively, massively parallel DNA sequencing technologies have revolutionized single nucleotide polymorphism (SNP) discovery and the study of genome variation of diverse categories [13]. 454 Life Sciences' pyrosequencing technology was the first next-generation sequencing (NGS) platform to reach the commercial market, offering relatively long reads and solutions to previous bottlenecks such as library preparation, template preparation and sequencing [14]. However, the ambiguous length of homopolymer runs, a primary limitation of this pyrosequencing-based method, may prohibit the sequencing of highly biased genomes.

High-resolution genome views provided by new sequencing technologies can be especially informative when applied to progeny clones derived from genetic crosses. Homologous recombination plays an essential role in ensuring correct chromosomal segregation during meiosis [15] and increases genetic diversity by reshuffling haplotypes; furthermore it can homogenize alleles through gene conversion [16]. In current models, meiotic recombination is initiated by formation of a double-strand break (DSB). The break is repaired through a series of steps, involving end resection, synthesis and ligation, using the homologous chromosome as a template [15]. Repair results in either a crossover (CO), i.e. reciprocal exchange accompanied by a tract subject to gene conversion, or a non-crossover (NCO), i.e. a tract subject to conversion but not associated with reciprocal exchange [2].

454 sequencing has been used to discover SNPs in a variety of organisms including plants [17-19], Rhesus Macaque [20], human [21] and bacteria [22]. This and other next-generation platforms redefine the quest for high-density marker discovery and genotyping, presenting an opportunity for obtaining a high resolution view of the genome to comprehensively link various types of allelic variants to phenotypes. For example, the 454 technology already has been applied to recombinant inbred lines of rice [6] and soybean [23]. Longer sequence reads combined with paired end reads will facilitate better mapping to reference genomes.

Here, we use the 454 Genome Sequencer FLX (GS FLX) platform for whole genome shotgun sequencing (WGS) to characterize the genomes of two *P. falciparum *progeny strains derived from a well-studied genetic cross between a multi-drug resistant and generally drug sensitive parent [24,25]. In addition to demonstrating the effectiveness of 454 WGS, we demonstrate the first high-resolution allele discovery method to monitor recombination events and their breakpoints along with other forms of genetic variation that distinguish these sibling parasite clones. We examine outcomes of meiosis that can only be recognized at nucleotide-level resolution, including genotype changes accompanying COs and NCOs that can refine our understanding of CO distribution or possible alternative double strand break resolution pathways in *P. falciparum*.

## Results and Discussion

**Figure 1:**
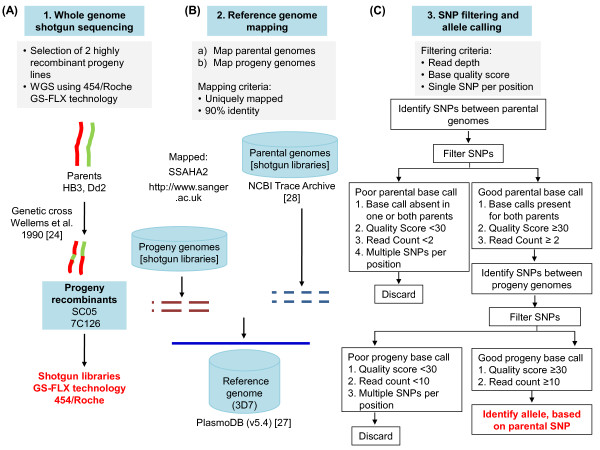
**Overview of genome resequencing and SNP allele determination**. (A) 454 GS FLX sequencing technology was used for shotgun genome sequencing to fine-map crossovers and other structural variants in 2 clonal progeny lines of *P. falciparum*. (B) WGS reads of the progeny genomes as well as the WGS reads from the parent genomes [28] were mapped to the common reference sequence (strain 3D7, PlasmoDB 5.4 [27]). (C) High quality SNPs (see methods) captured in parent and progeny genomes were used for allele screening.

High throughput sequencing is particularly suited for high-resolution marker discovery and linkage mapping [6,7]. We show that WGS sequencing using the 454 GS FLX sequencing platform is suitable for SNP allele detection even in the highly (A+T) biased malaria parasite, *P. falciparum*. By sequencing the 23 Mb genomes of two haploid progeny clones derived from a genetic cross at more than 30× coverage (Figure 1A), we captured high resolution information on crossovers, gene conversion and genetic variation within the progeny genomes 7C126 and SC05, relative to their parents.

### WGS pyrosequencing and *de novo *assembly

Two picotiter plates for each parasite line were sequenced on a GS FLX to generate 2,531,738 and 2,640,849 reads, comprising 766 and 828 Mb of sequence for 7C126 and SC05, respectively (Table 1). The GS FLX produced an average read length of approximately 300 bp for each genome. 3 kb paired end libraries were generated for each parasite and assisted with *de novo *assembly (Table 2). The largest contigs obtained for the two progeny were approximately 34 kb. The contigs were assembled into 970 and 2349 scaffolds, with an N50 scaffold length of 35.5 kb and 11.1 kb, for 7C126 and SCO5 respectively.

**Table 1:** Sequencing parameters of shotgun and 3 kb libraries of *Plasmodium falciparum *clones SC05 and 7C126 with the 454/Roche GS FLX sequencing platform.

Library	Number of sequencing plates	Number of Reads	Number of Bases	**Mean**^**1 **^**Length**	**Mode**^**2 **^**Length**
7C126 (Shotgun)	2	2,531,738	765,582,490	303	404
SC05 (Shotgun)	2	2,640,849	828,067,170	314	418
7C126 (3 kb)	½	438,749	118,727,308	271	298
SC05 (3 kb)	½	229,638	54,101,992	236	275

^1^Mean length - average length of reads in the library

^2^Mode length - read length occurring most often in the library

**Table 2:** Comparison of *de novo *assembly between 454 GS FLX, Sanger and Illumina platforms.

	**454 pyrosequencing**		**Sanger sequencing^**		Illumina sequencing*	
	
	**Progeny**		**Parents**		**Reference genome**	
	
	**7C126**	**SC05**	**Dd2**	**HB3**	**NP-3D7-S**	**NP-3D7-L**
	
Number of Scaffolds	970	2,349	2,837	1,189	NA	NA
N50 Scaffold Size (kb)	35.5	11.1	19.11	96.5	NA	NA
Number of Contigs	9,452	9,597	4,511	2,971	26,920	22,839
N50 Contig Size (kb)	3.3	3.3	11.61	20.62	1.5	1.6
Largest Contig (kb)	36.7	34.4	NA	NA	NA	NA
Number of assembled bases (Mb)	20.8	21.1	19.5	23.4	19.02	21.09
Average Coverage	33×	36×	7.8×	7.1×	43×	64×

^ Sanger technology - [50], http://www.broadinstitute.org

* Illumina technology - [26]http://www.sanger.ac.uk; NP:No-PCR libraries; Suffixes L and S indicate long and short sequencing runs performed from the same library

We compared the *de novo *assembly results from 454 GS FLX platform with the parental genome assembly information obtained using conventional Sanger technology, and 3D7 resequencing assembly information using the Illumina platform.

Given the concern that pyrosequencing may be fallible in a highly (A+T) biased genome, we compared *de novo *assembly parameters for these 454-derived progeny reads with the parental genome sequence derived from standard dideoxy-based sequencing (Table 2). We demonstrate that the GS FLX performed surprisingly well with this technically challenging genome and the increased throughput of this system affords the increased fold coverage needed for downstream applications, including genotyping and allele discovery. This study demonstrates that the higher read depth and genome coverage generated by 454 technology substantially improves the quality (e.g. confident SNP calls) and efficiency of high through put marker discovery than can be obtained using microsatellite markers and microarray derived single feature polymorphisms (SFP).

We compared the results of the 454 assembly data of the progeny genomes to that of the 3D7 genome assembly generated by Illumina technology [26] to assess the performance differences between the two NGS technologies in a highly (A+T) rich genome. While the standard library preparation method in Illumina technology did not permit *de novo *assembly, the improved no-PCR method enabled *de novo *assembly [26]; this was comparable to the *de novo *assembly statistics obtained using 454 technology, at approximately 36× coverage with considerably fewer contigs than that with the Illumina modified no-PCR method. Furthermore, we aligned contigs larger than 1 kb to the 3D7 assembly (nuclear DNA - PlasmoDB v 5.4 and apicoplast/mitochondrial reference sequences [27]) to search for segments that may be mis-assembled/missing in the current reference genome. No substantial regions were found to be missing from the current genome assembly. Only 14 of our contigs remained unaligned (6 contigs in 7C126 = 6.2 kb, and 8 contigs in SC05 = 8.9 kb), one 16 kb contig from each progeny sequence appeared to be contaminating human mitochondrial DNA. The remaining 12 contigs were < 2 kb.

### SNP detection and allele identification

To establish the platform for calling parental alleles inherited in the progeny clones, we developed a four step procedure: (1) map parental reads to the reference genome (3D7, PlasmoDB v 5.4, [27]); (2) identify SNPs between parents in these mapped regions; (3) map progeny reads to the reference genome; and (4) identify parental alleles in the progeny genomes (Figure 1B,C).

To identify SNPs between parents, we began by re-analyzing the trace reads of the parental genomes HB3 and Dd2 [28]. In step 1, we used strict criteria to call strain-specific SNPs by aligning the WGS trace reads of the Dd2 and HB3 strains to the reference genome. A total of 235,649 Dd2 reads and 243,509 HB3 reads aligned uniquely to the reference genome, covering 17.2 Mb (74.0%) and 21.3 Mb (92%) of the reference genome, respectively (Table 3, Additional file 1 summarizes the (A+T) content of the uniquely mapped regions). The difference in the mapped outcome of the two parental genomes could reflect sequence quality of the Dd2 WGS data or the high sequence variability present in the Dd2 genome compared to the reference genome, which in turn restrict the overlapping regions available for comparison between the parental genomes and subsequent selection of candidate positions for allele filtering. Occasionally, more than one base call was detected in a minor population of reads. Because these genomes are haploid, multiple alleles are not expected. These positions could represent sequencing errors, mapping errors, copy-number associated differences, or mutations arising during *in vitro *culture and are considered further below. For the purpose of marker selection, we excluded these positions with secondary alleles from further analysis.

**Table 3:** Mapping reads from progeny and parent genomes to the reference genome.

Method of Sequencing	Parasite Name	**Unique positions mapped**^**3**^	
		
		Number (bps)	Percentage
**Sanger**	HB3	21,326,115	91.7
	Dd2	17,226,364	74.0
**454-Roche**	7C126	21,600,531	92.8
	SC05	21,732,965	93.4

^3^Given as the percentage of the 3D7 genome coverage

All genomes had comparably similar distribution of the total bases sequenced (approximately 23 Mb) in terms of unique positions, however Dd2 had reads mapping to only 74% of the reference genome reducing the number of positions for screening alleles.

In step 2, we established a set of 24,599 high quality SNP markers by requiring uniquely mapping reads, with no mixed base calls at any SNP position, an average quality score of ≥ 30, and at least 2 reads supporting the base call identity (Additional file 2). Relaxed mapping criteria will increase the total number of SNPs detected but with the cost of decreased specificity.

For steps 3 and 4, we used the 454 GS FLX progeny clone sequence data to assess allelic variation and recombination in the context of available parental genome sequences. In step 3, a total of 1,738,923 reads from 7C126 and 1,802,733 reads from SC05 were uniquely aligned to the reference genome (Table 3). These covered 21.6 Mb positions of the reference genome (92.8%) in 7C126 and 21.7 Mb positions (93.4%) in SC05.

In step 4, each of the progeny strains (7C126 and SC05) was genotyped at the candidate SNP loci that distinguished the parents with the added requirement of at least 10 reads supporting the base call. Although these requirements reduced the sensitivity in detecting SNPs, especially in low coverage regions, it increased the specificity of true SNP detection by lowering the likelihood of including false variants that arise due to sequencing and/or mapping errors. We analyzed only SNPs and excluded all indels and variants involving more than one nucleotide. In parallel to the work presented here, our lab developed a gene chip to resequence 45,000 SNPs cataloged in PlasmoDB [27] (M.T. Ferdig, unpublished). Of the 24,585 SNPs identified in this study, 2,468 were encoded on the gene chip and produced identical base calls in 2,431 (98.5%) between the two platforms for clone 7C126. While we cannot discern at this point how much each platform contributed to the small disparity, we conclude that our accuracy in SNP calls is ≥ 98.5%.

**Figure 2:**
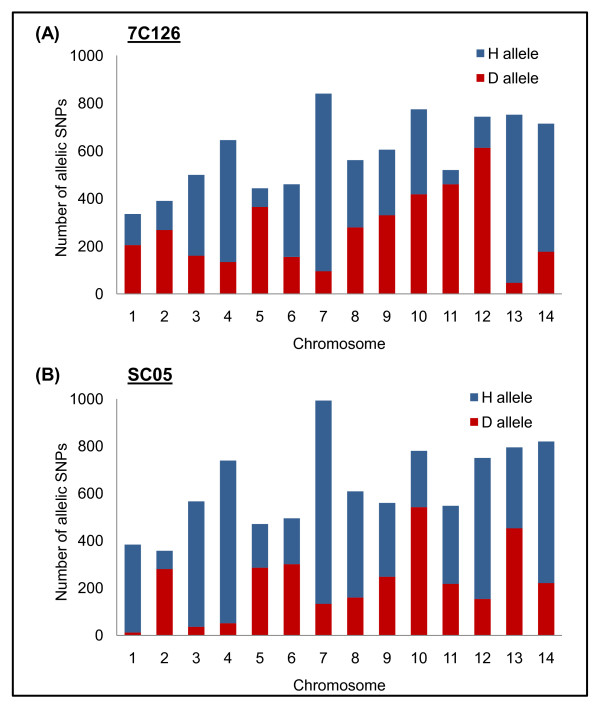
**Genome-wide allelic SNP distribution**. Summary of total bases used for allele calls (quality score ≥ 30, read number ≥ 10) and SNPs detected in each progeny (A-7C126, B-SC05). The progeny are predominantly HB3 parent like. For most positions a single base call was detected; however, mixed positions, i.e. alternate SNP positions were also detected (7C126-0.1%, SC05-0.09%). Of the high confidence single base calls, approximately 8,000 alleles were detected for each progeny line. Interestingly, approximately 0.001% of the SNPs were putative *de-novo *SNPs that differed from both parents.

**Figure 3:**
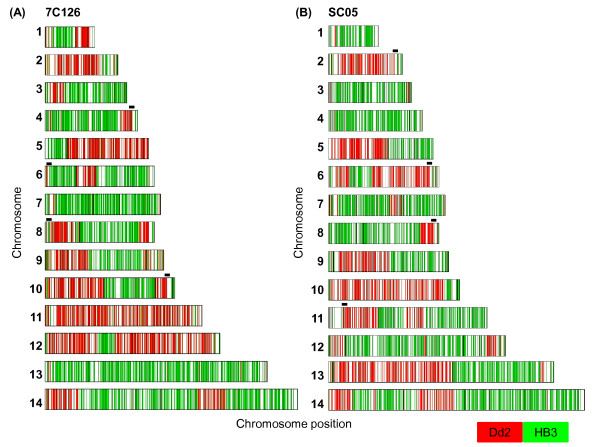
**Genome wide distribution of crossovers in progeny strain 7C126 and SC05**. Genome wide distribution of COs in (A) 7C126 and (B) SC05. Chromosomes 1-14 are lined up from top to bottom. Each line within a chromosome represents a single SNP marker. HB3 alleles are shown in green bars and Dd2 alleles are shown in red. 23 and 20 COs and putative gene conversion events were detected for 7C126 and SC05 respectively in the current MS marker based linkage map [25]. In contrast, the 454 SNP marker map detected 27 and 24 COs for 7C126 and SC05 respectively. The 454 SNP marker map was successful in the discovery of previously unknown CO events (highlighted with black bars). Refined views of the previously unknown COs are depicted in Additional file 3.

Of the total mapped positions, approximately 7 Mb of the genome in each progeny genome (7C126 = 6,971,934, SC05 = 7,408,764) was used for allele typing. Note that the numbers are lower than for each individual parent because only ~7 Mb met the stringent coverage and quality thresholds. Parental SNP identity was used to call the genotype at each allelic position in each progeny. A total of 8,201 SNPs were genotyped in 7C126, while 8,441 were genotyped in SC05 (Table 4, chromosome wide allele counts are given in Figure 2, the genome-wide allele distribution is given in Figure 3).

**Table 4:** Summary of high quality mapped base calls and allelic SNPs in progeny genomes

	Base calls			Allelic SNPs		
	**Total**	**Single**^**4**^	**Multiple**^**5**^	**Total Alleles**	**H Alleles**	**D alleles**

**7C126**	6,994,238	6,971,934	22,304	8,201	4,575	3,706
**SC05**	7,429,523	7,408,764	20,759	8,441	5,774	3,095

^4^Single base call per mapped position

^5^Multiple, alternative base calls per mapped position

### Genome-wide detection of COs and NCOs

**Figure 4:**
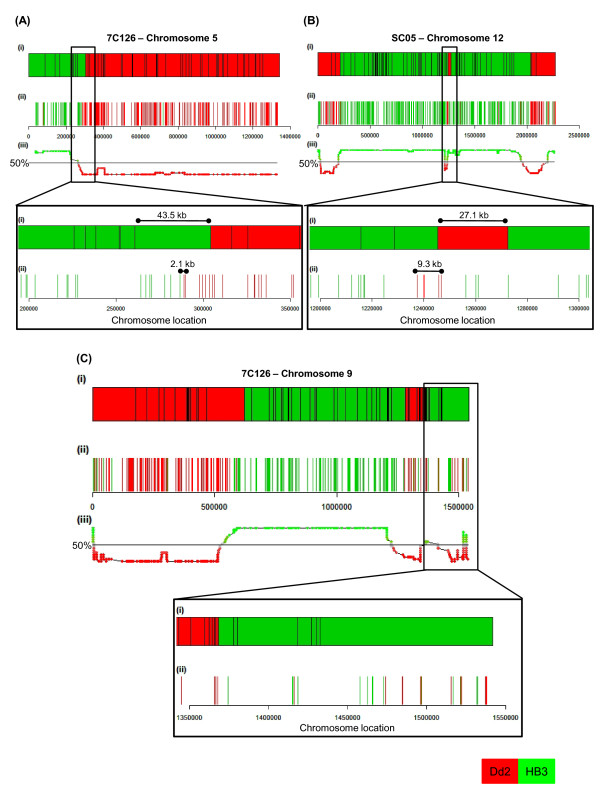
**CO and NCO gene conversion breakpoints**. A 15 SNP sliding window analysis was used for the detection of recombination breakpoints. (A) CO breakpoint in 7C126, (B) NCO gene conversion in SC05 and (C) complex CO breakpoint accompanied by a conversion tract (defined by rapid allele changes at/near breakpoint) in 7C126. Increased marker resolution refined the location of CO breakpoints (indicated by a double arrow). (i) MS markers in the *P. falciparum *linkage map [4]. (ii) SNPs detected by 454 sequencing. Each line represents a single SNP marker. (iii) Allelic ratio summary of the 15 SNP sliding windows is given in the plot by the first SNP position in each window. i, ii and iii were ordered according to the chromosomal location (PlasmoDB v5.4 [27]).

A sliding window including 15 contiguous SNPs was used to identify recombination breakpoints (adapted from Huang et al. [6], see Methods) (Figure 4). The method was modified to enable genome-wide detection of NCOs (see methods). The existing linkage map comprises 901 RFLP and microsatellite markers with approximately 25.5 kb average spacing. It identified 23 COs and single marker defined DCOs (identified as potential gene conversions) for 7C126 and 20 COs and single marker defined DCOs for SC05 [25]. The current study achieves an even higher marker density in the HB3 × Dd2 genetic cross with a resolution of approximately 1 marker every 3.3 kb, allowing for the detection for previously undetected COs. The current study identified 27 and 24 CO events for 7C126 and SC05 respectively (Figure 3) and enabled the discovery of previously unknown CO events (4 in each progeny; Additional file 3 shows refined views of Figure 3). In both progeny, with a single exception, each chromosome had at least one CO, consistent with the expectation of one obligate CO per pair of homologous chromosome [29]. Furthermore, 22 and 25 putative NCOs were detected in 7C126 and SC05, respectively. Three of the NCOs detected in this study corroborated single marker microsatellite events proposed by Su et al. to be gene conversions [25]. Most chromosomes carried one or more NCOs; however, NCOs were not detected on 5 chromosomes (7C126 - Chr 5 and 7, SC05 - Chr 1, 5, and 6). The conservative approach employed here to call NCOs in our study misses the smaller conversion events which may be an important source of genome variation.

Crossover breakpoint resolution depends on the SNP allele density as well as their distribution across the breakpoint region and the length of the conversion tract accompanying the CO/NCO breakpoint (Figure 4). It also depends on the sequence coverage in the region of the tract. The CO breakpoints occurred in a median breakpoint window of 88.5 kb (7C126, Minimum = 5.6 kb) and 101.8 kb (SC05, Minimum = 0.7 kb). Simple breakpoints, where one parental allele transitioned smoothly into the other parental allele (Figure 4A), and complex CO breakpoints, accompanied by a conversion tract with frequent allele changes (Figure 4C), were identified (Additional file 4). Most COs are simple, while a few COs (7C126 = 9/27, SC05 = 8/25) are associated with complex tracts. Mancera et al. [2] describe complex patterns of genotype changes in both COs (11.5%) as well as NCOs (3.4%). Such complex tracts were also observed by Qi et al.[30], and are consistent with the repair of heteroduplex DNA after Holliday junction formation and resolution [2,30].

**Figure 5:**
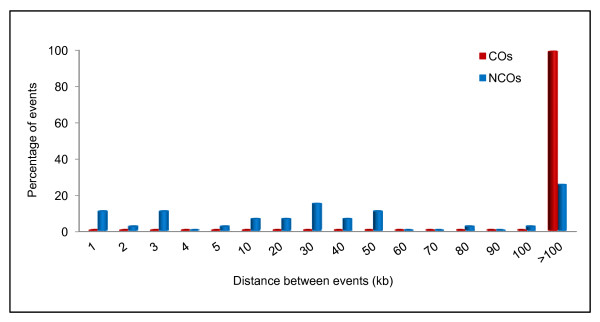
**Distance between predicted CO and NCO breakpoints**. Certain chromosomes were observed to have multiple COs (7C126 = 9, SC05 = 7) and NCOs (7C126 = 10, SC05 = 9). The distance between the beginning of the previous breakpoint window of an event and the start position of the consecutive breakpoint window of the next event was calculated. The distance between the different types of events were significantly different (P = 0.005). (A) NCOs were more commonly observed to occur within a shorter distance than COs. (B) When multiple COs occurred in a chromosome they were spaced > 200 kb apart.

The maximum CO and NCO lengths varied (Figure 5). As has been reported for fine structure studies in meiotic recombinant products of *Saccharomyces cerevisiae *[2,30], *P. falciparum *exhibits a wide range of COs and NCOs lengths. The distance between multiple COs in a chromosome was spaced > 200 kb apart, while this distance varied for NCOs (0.01 - 1,802 kb) (Figure 5). The distance between COs (as well as the distance between NCOs) could reflect interference mechanisms [29] in *P. falciparum*. CO interference which is a consequence of CO regulation has been associated with COs, but recent work has revealed that it is not inherent to all COs [31]. Further, recent work from genome-wide studies of COs and NCOs has shown interference not only between COs, but between COs and NCOs as well [2], highlighting the importance of comprehensive genome-wide analysis of COs and NCOs to address mechanisms of CO regulation. Mechanisms of CO/NCO generation, CO resolution, interference, and CO homeostasis are not well understood in *P. falciparum*. Elucidation of COs and NCOs, as well as mechanisms that regulate conversion tract length can reveal the frequency and extent of loss of heterozygosity within short distance linkage disequilibrium [2,30].

### Single nucleotide base variants

The ability to distinguish true sequence variants from sequencing errors is a fundamental challenge in the discovery of SNP variants and genotyping efforts; thus it is important to understand the types and probabilities of error in base calls [32]. Characteristic biases occur in sequence errors due to qualities of the queried base and the sequence context [33]. Technical issues specific to 454 technology include: nucleotide calling difficulties within homopolymers; sequencing failure arising from incomplete homopolymer extension; base mis-incorporation by residual nucleotides during the nucleotide flow step; mixed template beads, and overrepresented single templates distributed across different beads [34]. The haploid nature of the *P. falciparum *genome provides a unique opportunity to gain insight into systematic biases that may be introduced by 454 technology in an (A+T) rich genome. As a predominantly haploid organism, single nucleotide base variants differing in base composition from either of the parental sequence or heterozygous alleles for a given genome position is not expected. Two types of variant base calls were detected in the progeny; *de novo *SNPs and alternate SNP positions (i.e. multiple base calls per genome position).

*De novo *SNPs are defined here as bases in the progeny that are different from those of either parent. Eighty (0.0011%) and 128 (0.0017%) *de novo *SNPs were detected for 7C126 and SC05 respectively in high quality mapped base positions (i.e. single base call, ≥ 10 reads; average QS ≥ 30). The number of *de novo *SNPs detected is considerably lower than expected sequencing errors (estimated probability of an incorrect call = 0.1%) with a phred quality score of 30 [35].

Most *de novo *SNPs in the progeny (89% in 7C126 and 93% in SC05) occurred at positions that are identical in the parents. Eight loci were chosen and sequenced by traditional Sanger (capillary) methods in Dd2, HB3, SC05 and 7C126 (Additional file 5). For six of these, the progeny base was the same as a parent, indicating a 454 sequencing error. For the other 2 positions, the SNPs detected as *de novo *were concordant with a parent sequenced in our laboratory, indicating either differences between the clone used for original WGS and the clone of Dd2 strain used for resequencing in our lab, a sequencing error in the parental WGS sequence [8,10], or a sequence alignment error. This implies an error rate of 0.07/10 kb in high quality single allele SNPs.

Local sequence context affects genome sequence coverage as well as base call errors in NGS platforms [36]. To investigate the sequence characteristics connected with the *de novo *base calls, we searched for local sequence context, such as association with homopolymer tracts, because most 454 sequencing errors occur in homopolymer tracts [37,38] of 5 or more [39]. None of the positions analyzed were located in homopolymer tracts of > 5 bps (Additional file 6). Of all detected *de novo *base calls only 12 positions were found to be in homopolymer tracts of 3-5 bps (NNDN or NDNN, where N = nucleotide, and D = *de novo *SNP), indicating that these were not necessarily associated with homopolymer tracks; a majority (9/12) of the *de novo *SNPs detected in the homopolymer tracts were G (5/9) or C; suggesting these are unlikely to be sequencing errors arising due to homopolymer bias [40].

The probability of base substitutions occurring due to a sequencing error has been studied extensively [32]. Substitutions caused by sequencing errors are approximately 1-2 errors per 10 kb on the 454 platform [41]. Among the *de novo *SNPs, the proportion of transversion nucleotide substitutions (7C126 = 48% and SC05 = 56%) was greater than the proportion of transitions (7C126 = 46% and SC05 = 41%) in both progeny genomes. We observed a bias in the base changes (Additional file 7); T > A was the most common type of base conversion in both progeny. C > T, G > A transitions were frequent followed by transitions, A > G; T > C. The lack of SNP clustering coupled with the substitution biases may reflect Taq polymerase errors, and signal possible consequences of base mis-incorporation [40].

Both the parental genomes (derived from traditional Sanger based sequencing technology) and the two progeny genomes exhibited alternate SNP positions, i.e. multiple base calls at a single position. Of all positions uniquely mapped, the progeny clones sequenced in this study exhibited few positions 7C126 (0.1%) and SC05 (0.09%) with multiple base calls compared to the parental genomes. We observed no specific location bias for the genome-wide distribution of the alternate SNP positions in either parents or progeny genomes.

Alternate SNP positions can be expected in a haploid genome as a consequence of amplification artifact, sequencing errors, mis-mapped reads, or novel mutations occurring during *in vitro *culture. The primary base call of the alternate SNP positions was compared with the parental base calls at the position. The majority of the primary base calls were parental SNPs (approximately 80% of the allelic positions), while the majority of the secondary base calls were *novel *i.e. non parental SNPs (7C126 = 74%, SC05 = 86%). The *novel *primary base call position in 7C126 was primarily transitions, while it was primarily transversions in SC05. The uniformity of the distribution of alternate SNP positions and the type of substitutions observed in 7C126 suggested base conversion bias: C > T (shown in SC05 as well), G > A transitions were the most frequent followed by transitions, T > A, A > G, and T > C (Additional file 8); and suggest possible consequences of base mis-incorporation due to Taq polymerase errors [40]. The technology relies on single bead, single template amplification. Therefore amplification artifacts are rare, relative to actual sequence differences. Some pyrosequencing errors are also reported due to base miscalls arising from of mixed-template beads [34]. These alternate SNP positions could represent potential new mutations. Alternatively, in the case of a multi-clonal heterogenous population, there can be multiple independent high-quality reads with "normal" flowgrams which can give rise to alternate allelic positions. On the other hand, alternate allelic positions can also occur from paralogous sequences or repeats that are not present in the reference [42].

### Copy number variant detection

Comparative genomic hybridization (CGH) was used to detect the genome-wide distribution of large (> 1 kb) CNV regions in the two progeny clones. Five known CNVs that exist between parental strains were detected in the progeny (Additional file 9). These well known CNVs were used to investigate the possibility of using WGS read depth to detect CNVs. Read depth is consistent with known alleles except for chromosome 9, where smaller insertions and deletions are detected within a larger shared allele. As expected, no differences were found on the shared chromosome 2 deletions between the progeny (Table 5). The shared allele on chromosome 9 is also consistent, but may contain smaller insertions/deletions (significant windows = 13/52; chi square, p < 0.001). The chromosome 5 amplification was successfully differentiated between the progeny, consistent with the CNV breakpoints predicted by CGH. Chromosome 12 contains a small amplification in 7C126 and a larger amplification in SC05. The 7C126-specific amplification is highly significant with read depth window analysis and all but one neighboring region is significant in SC05. This could be an indication of the differences in the amplification profile between the two progeny or it may be a reflection of a lack of substantial sequence coverage in SC05. We conclude that 454 read mapping is accurate and could be used to detect copy number differences in these progeny.

**Table 5:** Five known copy number differences detected between parental strains using WGS read depth.

Chromosome	Type	No. of 2.5 kb windows	Significant windows (p < 0.001)	Highest Chi-square value
2	Deletion - in both	15	0	3.99
5	Amplification - 7C126	33	33	1212
9	Deletion - in both	52	13	50.6
12	Amplification - 7C126	1	1	85.44
12	Amplification - SC05	64	63	356.03

Read alignment density was consistent with the known CNV alleles except for chromosome 9, where smaller insertions and deletions are detected within a larger shared allele.

## Conclusions

Comparison of sequence data from the 454 GS FLX platform with genome sequence generated by conventional dideoxy-based sequencing demonstrates that the GS FLX data is favorably comparable to standard dideoxy-based sequencing for *de novo *assembly of an AT rich genome because the assembly statistics were similar to those of the parental genomes. The high throughput SNP marker detection method using 454 technology substantially improved the efficiency of allele discovery and crossover detection compared to traditional markers (i.e. MS and RFLPs) used in linkage analysis. By sequencing the 23 Mb genomes of two haploid progeny clones derived from a genetic cross at more than 30× coverage, this investigation captured high resolution information on COs, NCOs and genetic variation within the progeny genomes. Our approach for surveying recombination in this predominantly haploid genetic system allow for not only genome wide detection and fine scale analysis of recombination products but also reveal potential details on CO interference and double strand break resolution.

## Methods

### Parasites, DNA Extraction, and Microsatellite genotyping

*Plasmodium falciparum *strains 3D7, Dd2, HB3, 7C126 and SCO5 were thawed from genotyped source stocks and cultured at parasitemia suitable for DNA extraction. Parasites were grown at 37°C and 5% hematocrit in O+ human red blood cells using RPMI 1640 (Invitrogen, Carlsbad, CA) supplemented with 0.5% Albumax I (Invitrogen), 0.25% sodium bicarbonate (Mediatech, Inc., Manassas, VA) and 0.01 mg ml-1 gentamicin (Invitrogen) under an atmosphere of 90% nitrogen, 5% oxygen, and 5% carbon dioxide. Cultures were gassed every day, the media was changed every 2 days and parasitemia was maintained below a level of 5%. Total genomic DNA was isolated from frozen culture using standard phenol-chloroform extraction. Each parasite DNA was genotyped for a set of 8 microsatellite markers to ensure clonality and to confirm parasite identities.

### Library production and shotgun sequencing

GS FLX Titanium shotgun libraries were made from genomic DNA according to the manufacturer's specifications at 454 Life Sciences (454 Life Sciences, Branford, CT). Briefly, sequencing was performed according to GS FLX standard protocols with the following modifications: due to the high (A+T) content of the *P. falciparum *genome, the concentration of thymidine in the sequencing reaction was increased to 1.4 times the recommended amount, and 150 cycles of sequencing were performed instead of the standard 100 cycles. Two GS FLX Titanium paired-end libraries (3 kb) were constructed and sequenced at 454 Life Sciences according to the manufacturer's specifications (454 Life Sciences, Branford, CT).

### Read Mapping

This SNP analysis scheme began with a comprehensive re-analysis of the trace reads of HB3 and Dd2 from the database [28] before attempting to identify SNPs. We obtained read and quality sequences for the Dd2 and HB3 strains from the NCBI Trace Archive in May 2009. The reads from the Dd2 and HB3 strains were computationally trimmed using LUCY [43] (parameters -error 0.05 0.50 -window 50 0.05 -bracket 10 0.10). The trimmed reads were aligned to the 3D7 reference assembly using SSAHA2 version 2.3.0.1 (-tags 1 -output cigar -diff 0 -identity 90.0 -best 1). The alignments were filtered using ssaha cigar with default parameters. Custom perl scripts were used to summarize the base call and quality information for all reads that map to each position of the reference genome. For each base call that occurs at a position, the coverage (number of reads) and quality scores are stored in a text file similar to the vertical multiple alignment (VMA) format [44]. The base call with the most reads is considered the primary base call. If a second base is called with two or more supporting reads, then it is stored as a secondary base call.

The 454 reads from the 7C126 and SC05 strains were aligned to the 3D7 reference assembly using SSAHA2 (-tags 1 -output cigar -diff 0 -identity 90.0 -best 1). The alignments were filtered using ssaha_cigar with default parameters. The primary and secondary base calls and quality scores were summarized into VMA in a similar manner as the parental strains.

### SNP calls and SNP verification

Parental SNP identification: The base calls and quality values of the sequence from the Dd2 and HB3 strains were considered at each position of the reference genome. We required each parent to have two or more reads with an average quality score of at least 30. Additionally, we required both parents to uniformly exhibit a single base at a position (no secondary base call). Positions that met all of these criteria were considered candidate positions for progeny genotyping, i.e. the Dd2 base call differs from the HB3 base call. The base calls and quality values of the 7C126 and SC05 strains were considered at each position determined above. We required a strain to have 10 or more read depth coverage with average quality score of at least 30. Additionally, to call an allele for a progeny clone, we required it to uniformly exhibit a single base at the position (no secondary base call). Positions that met all of these criteria were considered valid.

To further confirm the SNP calls, we compared the SNP calls on an independent platform. In parallel to the work presented here, our lab developed a gene chip to resequence 45,000 SNPs cataloged in PlasmoDB [27] (M.T. Ferdig, unpublished). Of the 24,585 SNPs identified in this study, 2,468 were encoded on the gene chip for direct comparison with 7C126 between the platforms.

### Allele calls

At each valid position that we identified as a parental SNP, we classified each strain as inheriting the Dd2 or HB3 allele, or alternatively, a *de novo *allele. To validate the *de novo *SNPs detected, we PCR amplified eight 1 kb regions overlapping *de novo *SNP loci. Each amplicon was sequenced bi-directionally (forward and reverse) using standard dideoxy-based sequencing on an ABI 3730xl DNA Analyzer. Sequencing chromatograms were analyzed with Contig Express (Vector NTI Advance™ software, Life Technologies Corporation, Carlsbad, CA).

### Recombination breakpoint prediction and verification

A sliding window approach was used for the prediction of recombination breakpoints [7]. The filtered single allele calls were assessed in 15 SNP intervals. Allele frequencies for each bin were calculated. A CO was predicted when the allele frequency in a window transitioned from one allele type to another (100% allele frequency). NCOs were defined as a locus consisting of opposite allele configuration within a larger surrounding region; and predicted with strict criteria: must contain at least 3 contiguous SNPs in the opposite allele configuration of the surrounding locus and must also include 8 of such SNPs in a 15 SNP window. This method will miss smaller NCOs involving < 3 contiguous SNPs.

The distance between events was calculated as the distance between the beginning of the previous breakpoint window of an event and the beginning of the consecutive breakpoint window of the next event. The chromosomal alignments at CO and NCO regions were visualized using Integrative Genomics Viewer [45]. Regions at CO and NCO regions were visually inspected in comparison with the parental genomes, for quality of read alignment and SNP distribution.

### Analysis of single nucleotide base variants

Custom perl scripts were used to analyze single nucleotide base variants including *de novo *SNPs and alternate allelic positions. *De novo *SNPs were defined as called bases in the progeny that are different from those of either of the parents. All *de novo *SNPs were checked for association with homopolymer tracts of > 5 bps (NDNNNN or NNDNNN or NNNDNN or NNNNDN where N = nucleotide, and D = *de novo *SNP) and 3 to 5 bps (NNDN or NDNN). Alternate allelic positions were defined for parental genomes as well as the progeny genomes. The base with the most reads was considered the primary allele, while the alternate base was considered the secondary allele at that position. Two different sets of read cutoffs were used to differentiate the secondary allele in parent (at least 2 supporting reads) and progeny (at least 5 supporting reads). Both *de novo *SNPs and the primary allele in progeny were analyzed for base substitution changes in comparison with the parental base using custom perl scripts.

### Large structural event detection

A custom 385 k NimbleGen array designed for the *P. falciparum *3D7 reference genome (PlasmoDB [27], 2006) using the standard CGH probe design protocol [46] was used [11]. The array comprises 385,585 probes semi-tiled across the genome at a 4 bp interval spacing with a minimum probe length of 45 bp, and a maximum length of 85 bp. Labeling and hybridization was carried out according to the standard NimbleGen CGH protocol [46]. 7C126 and SC05 were hybridized with reference 3D7. DNA fragmentation, labeling, hybridization, washing, and scanning were carried out using the standard NimbleGen CGH protocol, at the Genomics Core Facility (University of Notre Dame, Notre Dame, IN). The microarrays were hybridized and washed in a NimbleGen Hybridization System 4 (NimbleGen Systems, Inc., Madison, WI). Images were acquired by using The NimbleGen MS 200 Microarray Scanner (NimbleGen Systems, Inc., Madison, WI) at a 5 μm resolution. Probe intensity values were extracted from scanned images using NimbleScan extraction software (NimbleGen Systems, Inc., Madison, WI). The Cy3 and Cy5 signal intensities were normalized according to standard Nimblegen protocol (http://www.nimblegen.com/products/lit/cgh_userguide_v6p0.pdf). The normalized values were used for calculation of log_2 _ratio values and used for CNV detection using a segmentation model based on a Gaussian framework [47].

### CNV detection with read depth analysis

Five characterized copy number differences were used to test structural variation detection with 454 shotgun read library in the (A+T) biased genome. Read locations along chromosomes were derived from CIGAR alignments (see section iii Read Mapping) used for SNP discovery; reads were assigned to non-overlapping 2.5 kb intervals if at least 85% of its length aligned to that interval. To compute CNVs, we used a simple 2 × 2 Chi square test. We compared the proportion of reads in each non-overlapping 2.5 kb interval relative to all reads that mapped to all other intervals on the chromosome; and compared each window between each progeny. The resulting statistic was converted to a p-value based on a Chi square distribution with two degrees of freedom, but not corrected for multiple comparisons. This computational approach is more similar to array-based detection and digital expression (e.g., Man et al., 2000 [48]) than more traditional read depth approaches (e.g., Bailey et al., 2002 [49]) and was chosen to detect large (5 kb or larger) structural variation known to occur in the progeny genomes.

## Abbreviations

GS FLX: Genome Sequencer FLX; WGS: whole genome shotgun; CO: crossover; NCO: non-crossover; NGS: next generation sequencing; DSB: double strand break; SNP: single nucleotide polymorphism; QS: quality score; indel: insertion or deletion; CGH: comparative genomic hybridization; CNV: copy number variant; VMA: vertical multiple alignment; NCBI: National Center for Biotechnology Information.

## Authors' contributions

SU, BD, JT and MTF conceived the project. SU, AR, AT, SE and MTF conducted the read mapping and data analysis. SU grew parasites, extracted, genotyped DNA and carried out the CGH hybridizations. SU and BC performed ABI sequencing for SNP verification. Shot gun and paired end library preparation and 454 sequencing were carried out by BD along with library quality analysis and *de novo *genome assemblies. SU, AR, BD, JT, SE and MTF wrote the manuscript. All authors have read and approved the final manuscript.

## Supplementary Material

Additional file 1 (A+T) content of WGS reads in uniquely mapped regions of parents and progeny Click here for file

Additional file 2 SNPs between HB3 and Dd2 Click here for file

Additional file 3 **Refined views of previously unknown COs shown in Figure **3. Previously unknown COs detected in the progeny lines (highlighted with black bars in Figure 3) are indicated by double arrows in the chromosomal view (top) and zoomed-in view (boxed, bottom). (A-D) previously unknown COs in 7C126, (E-H) previously unknown COs in SC05. SNP map by 454 sequencing is presented in comparison with the MS marker linkage map in *P. falciparum *[4]. Each line represents a single SNP marker. HB3 alleles are shown in green bars and Dd2 alleles are shown in red. Click here for file

Additional file 4 **Visual inspection of mapped WGS sequence at CO and NCO gene conversion breakpoints shown in Figure **4. (A) simple CO breakpoint in 7C126, (B) NCO gene conversion in SC05 and (C) complex CO breakpoint accompanied by a conversion tract (defined by rapid allele changes at/near breakpoint) detected in 7C126. Chromosomal alignments at CO and NCO regions were visually inspected in comparison with the parental genomes, using Integrative Genomics Viewer [45]. (i) SNPs detected by 454 sequencing are ordered according to the chromosomal location (PlasmoDB v5.4 [27]). Each line represents a single SNP marker. HB3 alleles are shown in green bars and Dd2 alleles are shown in red. (ii) Comparison of SNPS and read alignments at selected SNP loci (arrow). SNPs are highlighted in blue [C], red [T], green [A] and brown [G]. Click here for file

Additional file 5 Resequencing results of *de novo *SNP positions Click here for file

Additional file 6 **Distance between consecutive *de novo *SNPs**. The distance between consecutive *de novo *SNPs were calculated to detect SNP clustering characteristic of sequencing errors or mis-mapping errors. 35% of the de novo SNPs were clustered in distances of less than 5 bps. Click here for file

Additional file 7 **Base conversion trends in *de novo *SNPs**. The type of base conversion was investigated for positions at which the parental base calls were identical. More transversions were detected for SC05 compared to 7C126 (A), but did not show a predominant base conversion bias from 7C126 (B). Click here for file

Additional file 8 **Alternate SNP positions**. The alternate SNP positions were assessed for their primary and secondary positions base call identity. Most primary base calls reflected the parental base call. The secondary base call position varied in the 2 progeny genomes in base call identity. Majority of the secondary base calls were parental in 7C126, whereas majority of the secondary base calls were non-parental in SC05 (A). Majority of the primary base calls were transitions in 7C126, while they were transversions in SC05 (B, C). Click here for file

Additional file 9 **Selected CNVs in 7C126 and SC05**. Comparative genomic hybridization (CGH) was used to detect large (> 1 kb) CNV regions in 7C126 (A) and SC05 (B). Five known CNVs that exist between parental strains were detected in the progeny in Chr 2, 5, 9 and 12. Click here for file
