# Supplementary material for: High-throughput 454 resequencing for allele discovery and recombination mapping in Plasmodium falciparum
Source: BMC Genomics. 2011 Feb 17;12:116. doi: 10.1186/1471-2164-12-116 (PMC3055840; doi:10.1186/1471-2164-12-116)
Supplement: Additional file 1 — (A+T) content of WGS reads in uniquely mapped regions of parents and progeny [file 1471-2164-12-116-S1.PDF]

Additional file 1 – (A+T) content of WGS reads in uniquely mapped regions of parents and progeny

Mapped positions	Sanger technology		454 GS FLX technology	
	HB3	Dd2	7C126	SC05
**A (%)**	40.2	39.5	37.4	37.5
**T (%)**	40.1	39.5	37.5	37.5
**C (%)**	9.8	10.5	12.5	12.4
**G (%)**	9.9	10.6	12.6	12.5
