# Supplementary material for: High-throughput 454 resequencing for allele discovery and recombination mapping in Plasmodium falciparum
Source: BMC Genomics. 2011 Feb 17;12:116. doi: 10.1186/1471-2164-12-116 (PMC3055840; doi:10.1186/1471-2164-12-116)
Supplement: Additional file 3 — Refined views of previously unknown COs shown in Figure 3. Previously unknown COs detected in the progeny lines (highlighted with black bars in Figure 3) are indicated by double arrows in the chromosomal view (top) and zoomed-in view (boxed, bottom). (A-D) previously unknown COs in 7C126, (E-H) previously unknown COs in SC05. SNP map by 454 sequencing is presented in comparison with the MS marker linkage map in P. falciparum [4]. Each line represents a single SNP marker. HB3 alleles are shown in green bars and Dd2 alleles are shown in red. [file 1471-2164-12-116-S3.PDF]

(A)

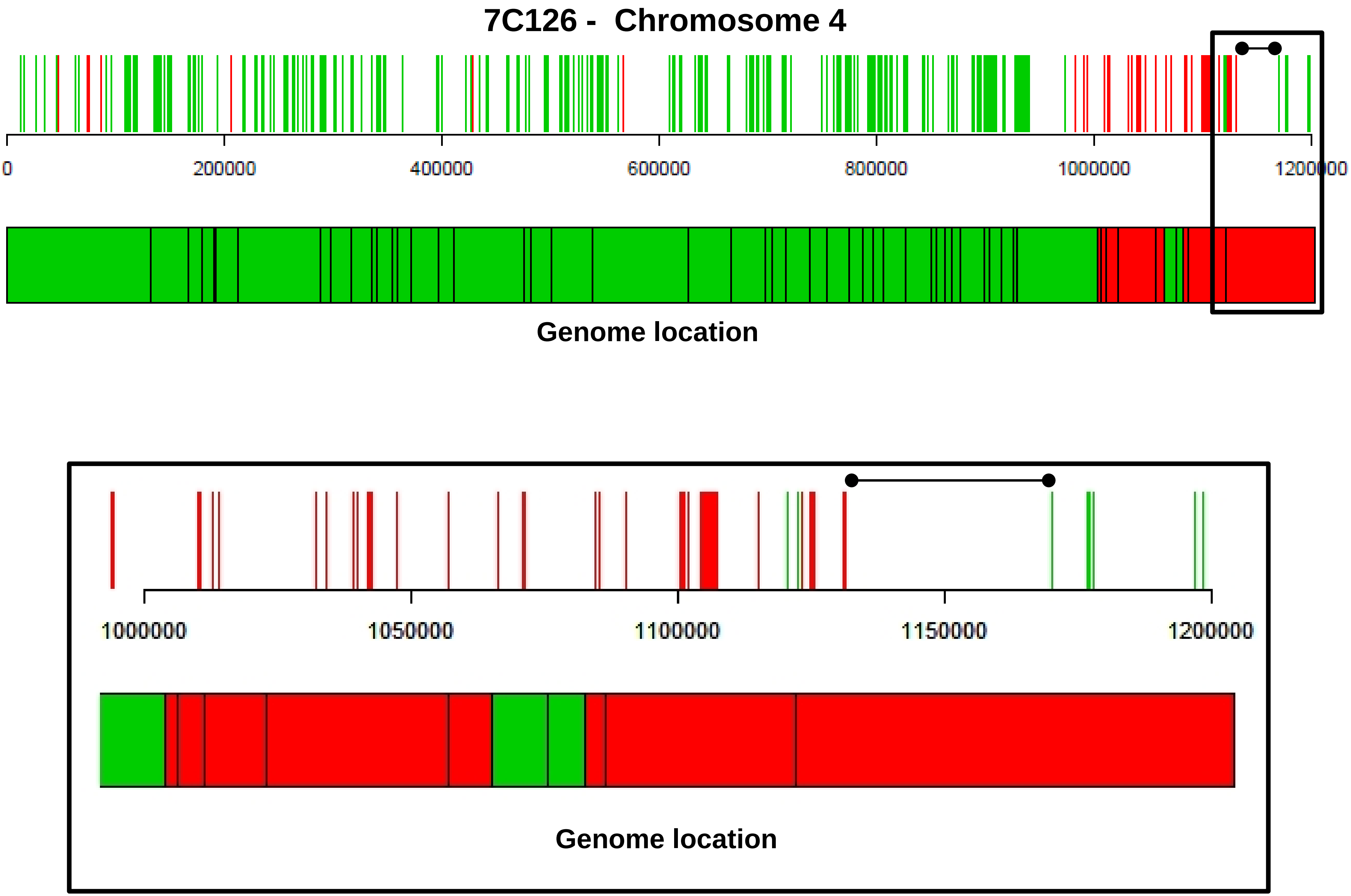
7C126 - Chromosome 4
0
200000
400000
600000
800000
1000000
1200000
Genome location
1000000
1050000
1100000
1150000
1200000
Genome location

(B)

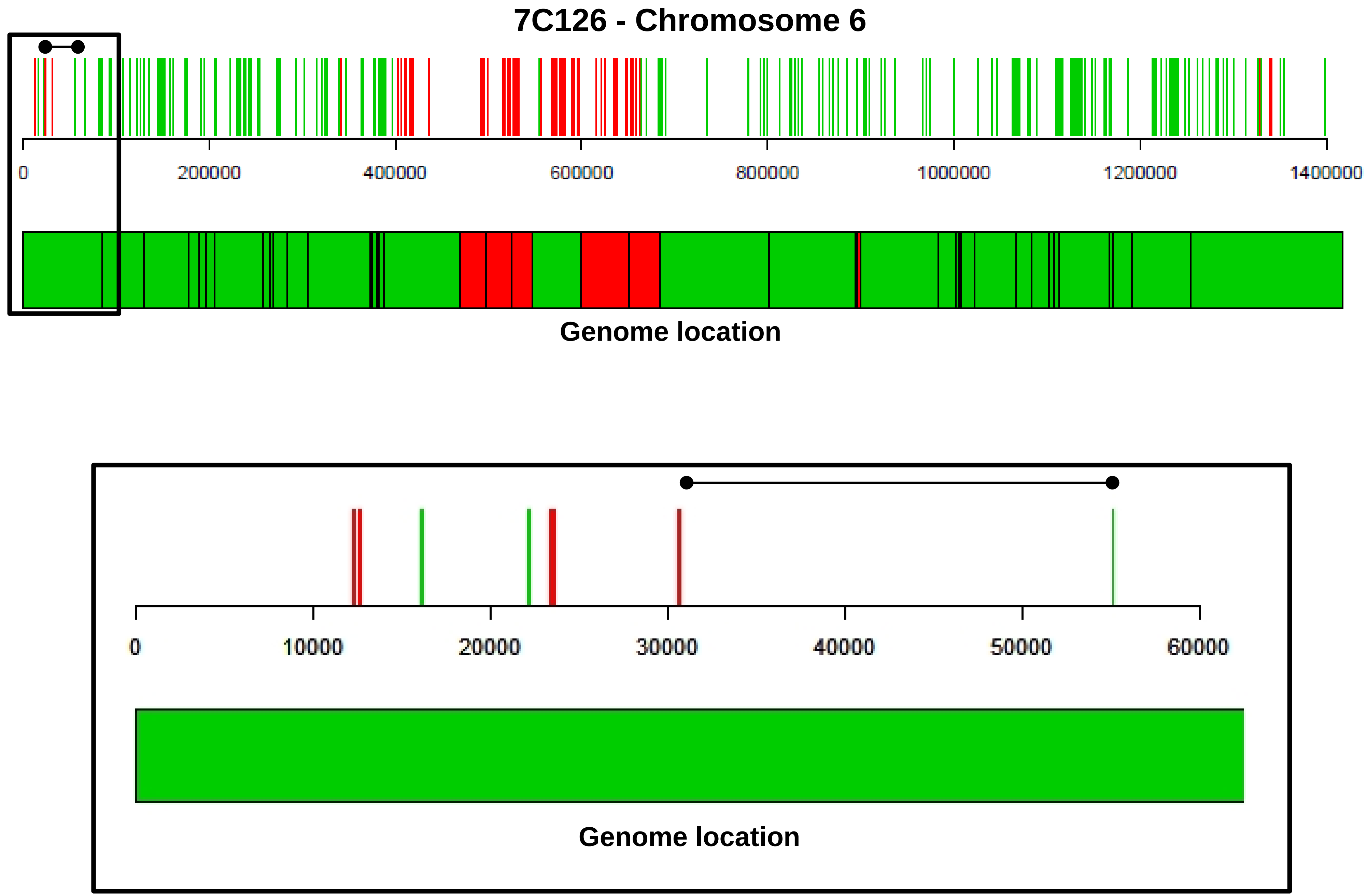
7C126 - Chromosome 6
0
200000
400000
600000
800000
1000000
1200000
1400000
Genome location
0
10000
20000
30000
40000
50000
60000
Genome location

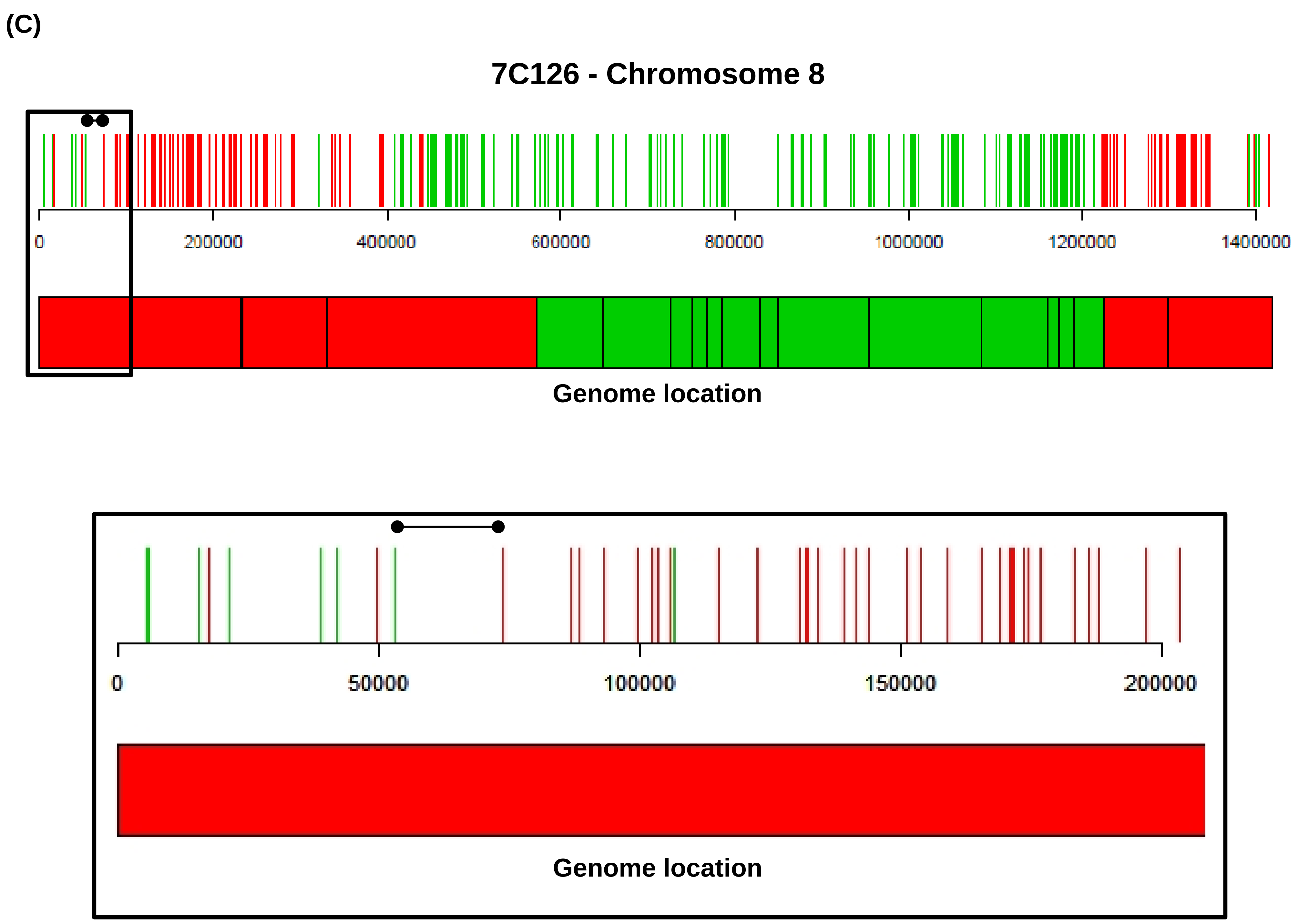
(C)
7C126 - Chromosome 8
0
200000
400000
600000
800000
1000000
1200000
1400000
Genome location
0
50000
100000
150000
200000
Genome location

(D)

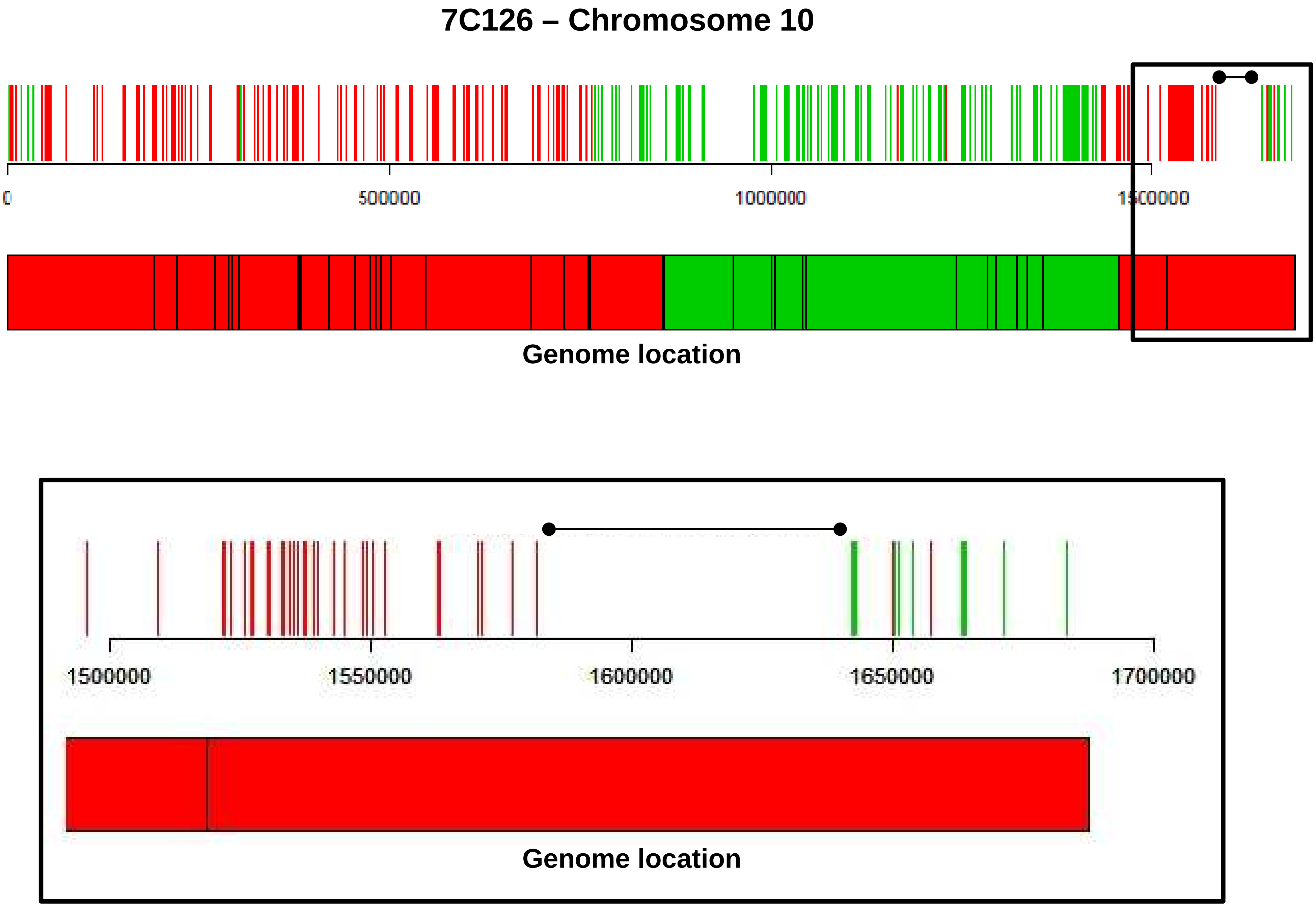
7C126 – Chromosome 10
0
500000
1000000
1500000
Genome location
1500000
1550000
1600000
1650000
1700000
Genome location

(E)

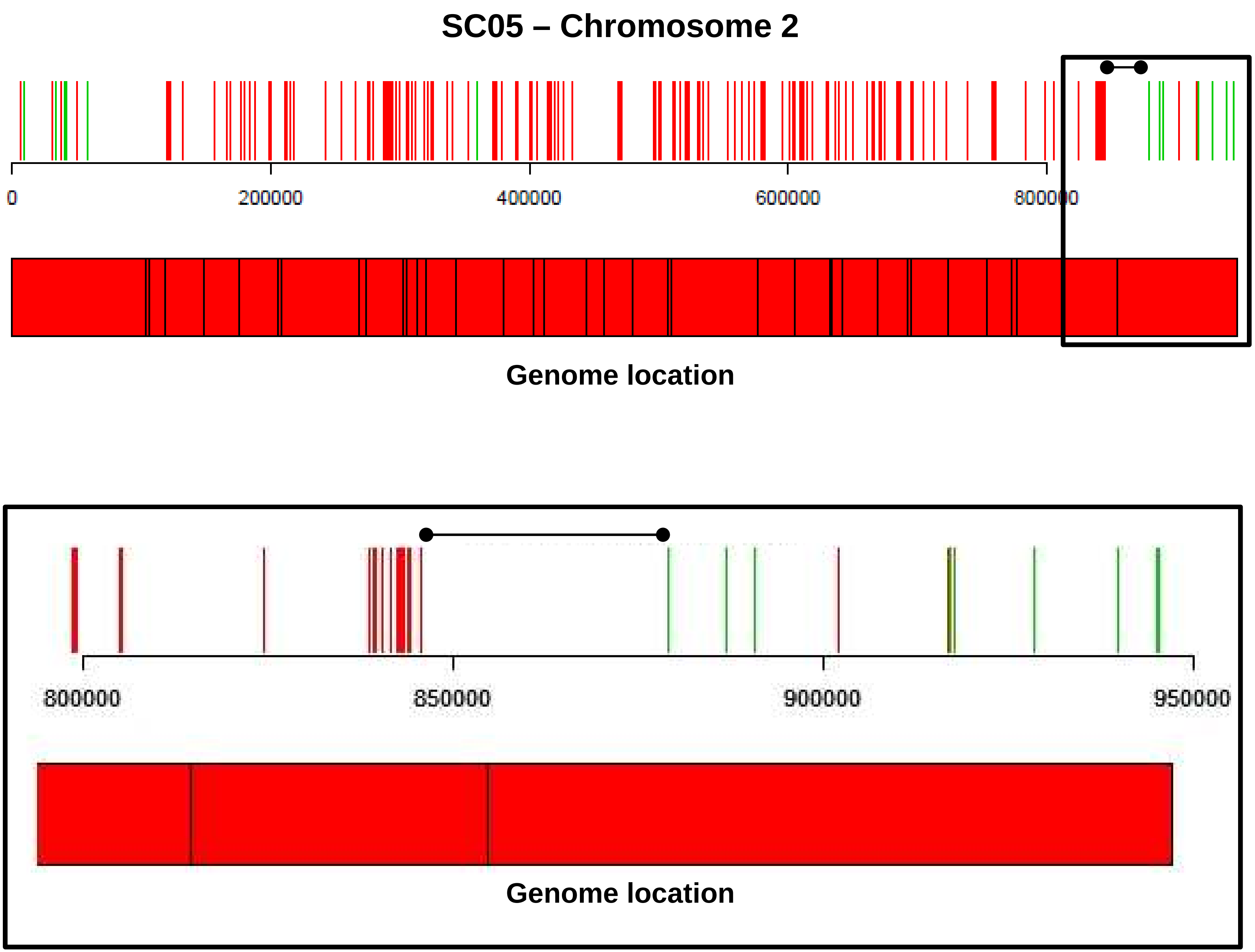

SC05 – Chromosome 2
0
200000
400000
600000
800000
Genome location
800000
850000
900000
950000
Genome location

(F)

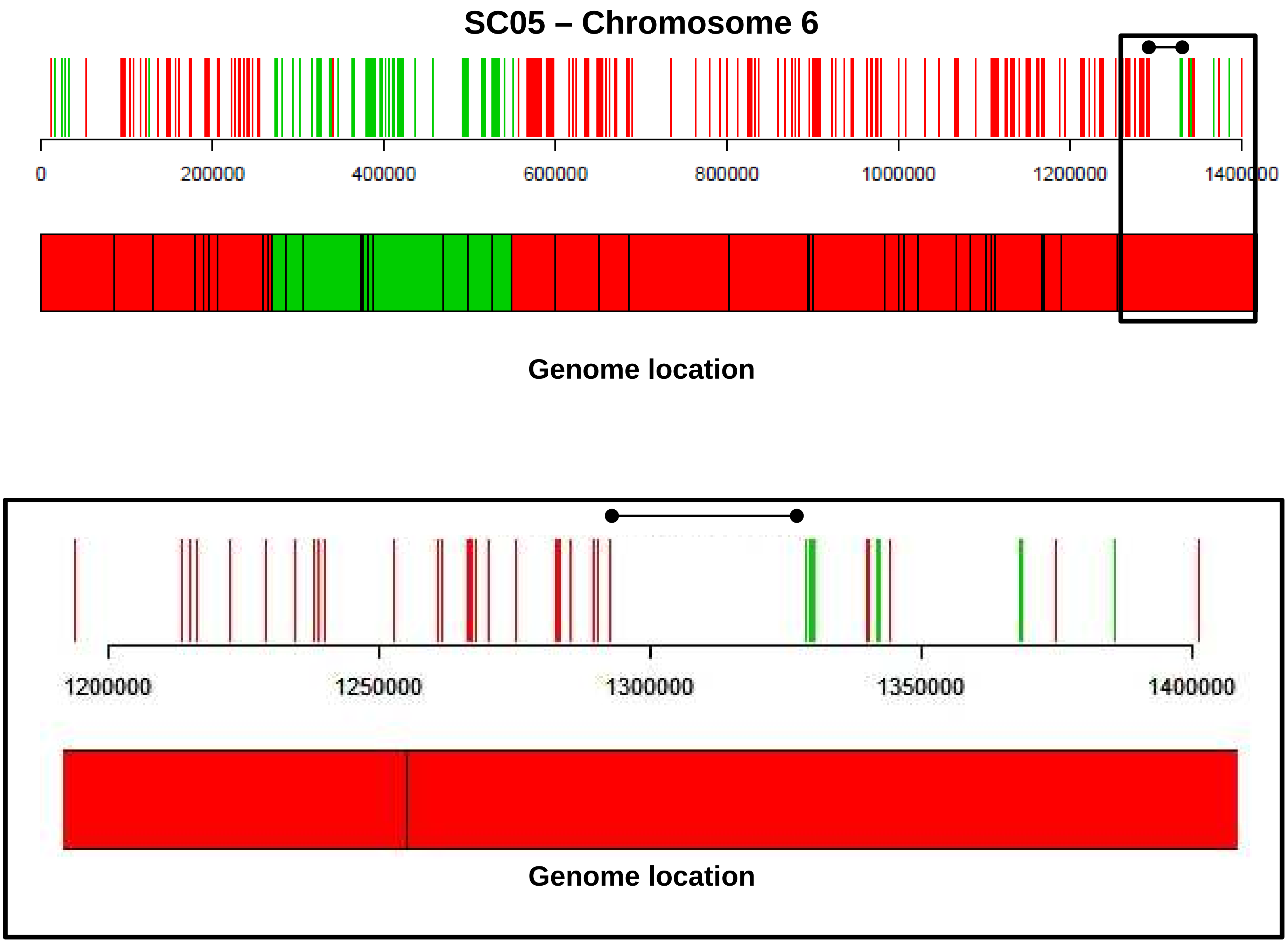
SC05 – Chromosome 6
0
200000
400000
600000
800000
1000000
1200000
1400000
Genome location
1200000
1250000
1300000
1350000
1400000
Genome location

(G)

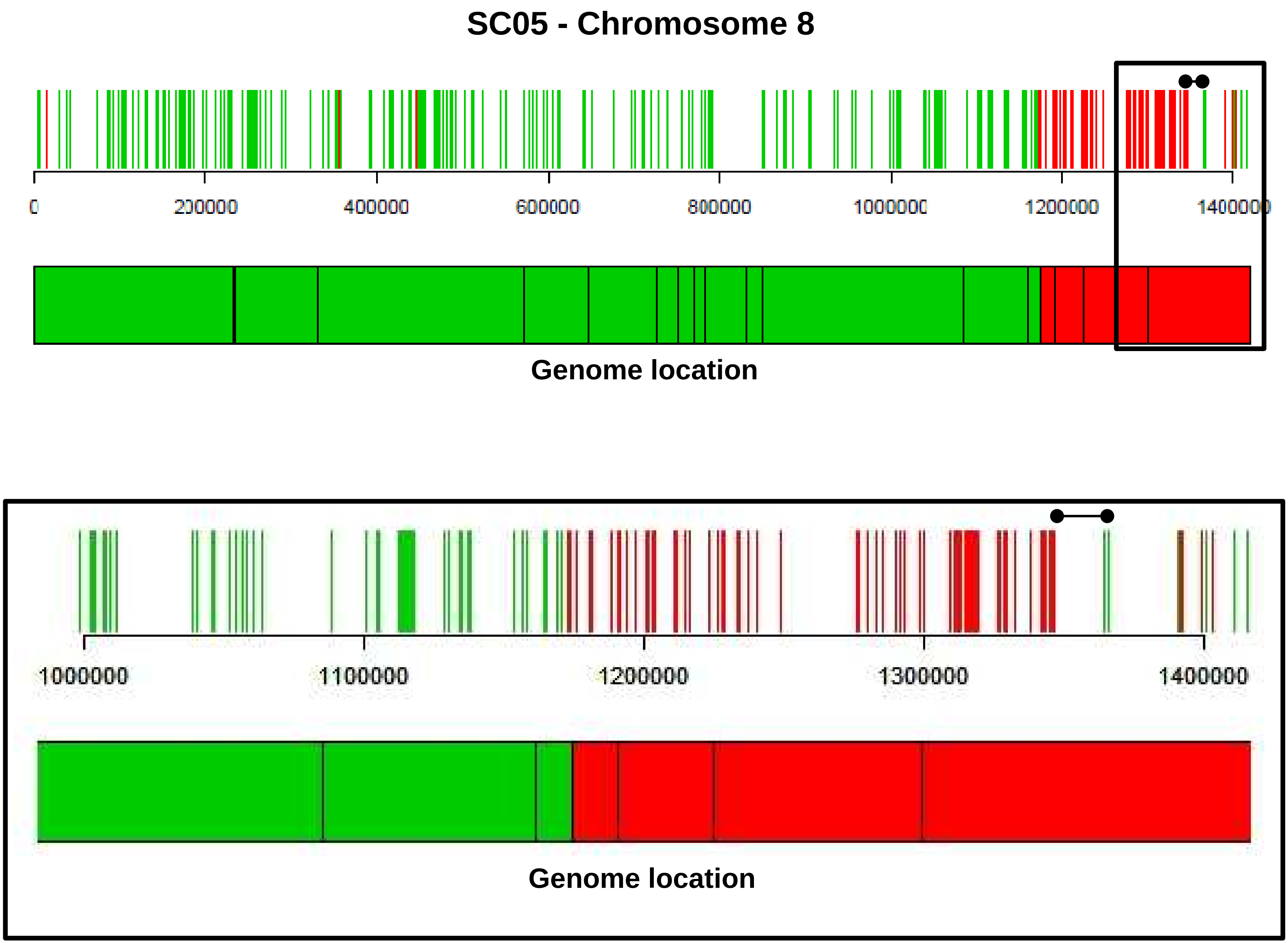
SC05 - Chromosome 8
0
200000
400000
600000
800000
1000000
1200000
1400000
Genome location
1000000
1100000
1200000
1300000
1400000
Genome location

(H)

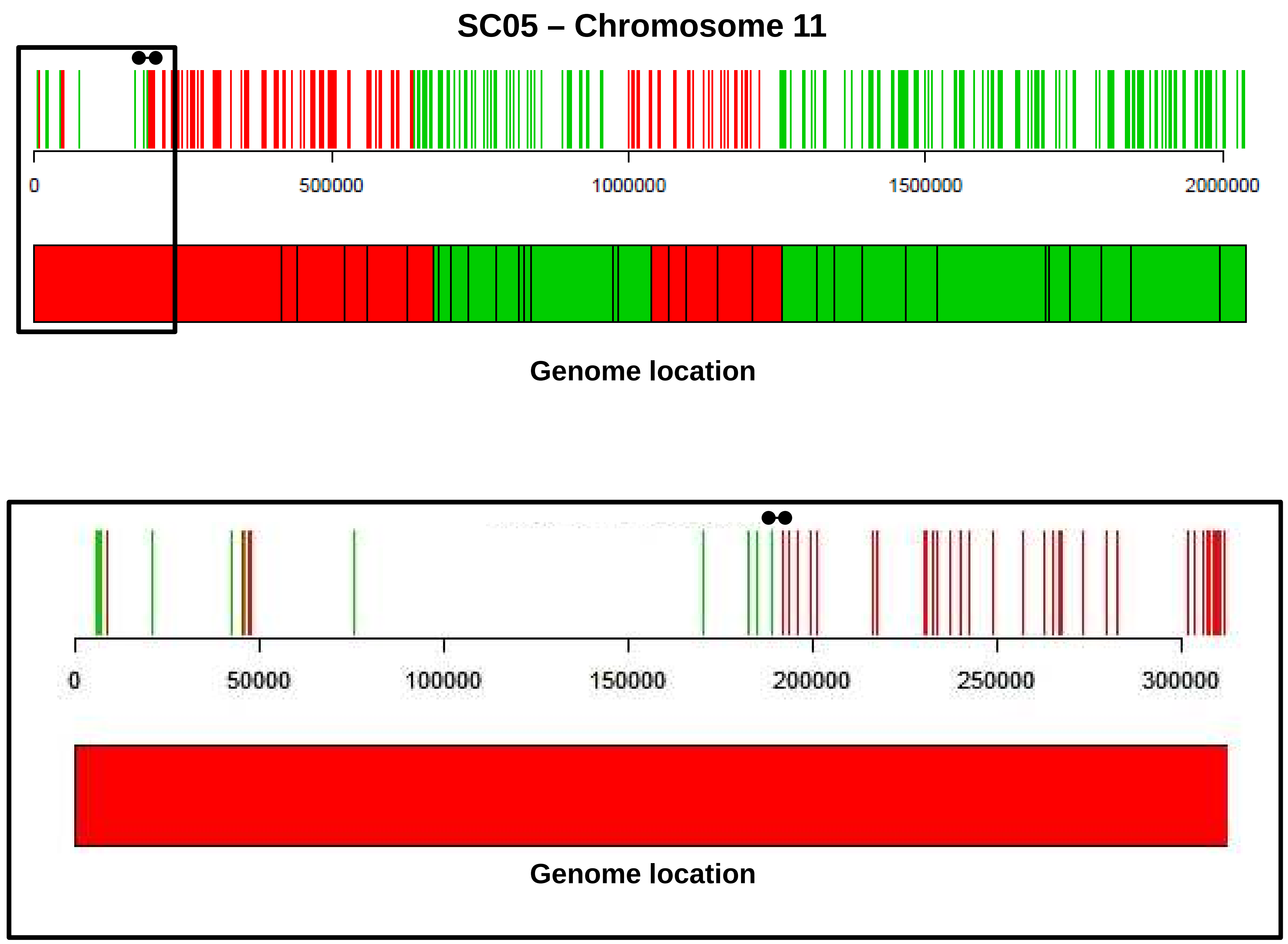

SC05 – Chromosome 11
0
500000
1000000
1500000
2000000
Genome location
0
50000
100000
150000
200000
250000
300000
Genome location

Additional file 3 - Refined views of previously unknown COs shown in Figure 3.

Previously unknown COs detected in the progeny lines (highlighted with black bars in Figure 3) are indicated by double arrows in the chromosomal view (top) and zoomed-in view (boxed, bottom). (A-D) previously unknown COs in 7C126, (E-H) previously unknown COs in SC05. SNP map by 454 sequencing is presented in comparison with the MS marker linkage map in *P. falciparum* [4]. Each line represents a single SNP marker. HB3 alleles are shown in green bars and Dd2 alleles are shown in red.
