# Supplementary material for: High-throughput 454 resequencing for allele discovery and recombination mapping in Plasmodium falciparum
Source: BMC Genomics. 2011 Feb 17;12:116. doi: 10.1186/1471-2164-12-116 (PMC3055840; doi:10.1186/1471-2164-12-116)
Supplement: Additional file 4 — Visual inspection of mapped WGS sequence at CO and NCO gene conversion breakpoints shown in Figure 4. (A) simple CO breakpoint in 7C126, (B) NCO gene conversion in SC05 and (C) complex CO breakpoint accompanied by a conversion tract (defined by rapid allele changes at/near breakpoint) detected in 7C126. Chromosomal alignments at CO and NCO regions were visually inspected in comparison with the parental genomes, using Integrative Genomics Viewer [45]. (i) SNPs detected by 454 sequencing are ordered according to the chromosomal location (PlasmoDB v5.4 [27]). Each line represents a single SNP marker. HB3 alleles are shown in green bars and Dd2 alleles are shown in red. (ii) Comparison of SNPS and read alignments at selected SNP loci (arrow). SNPs are highlighted in blue [C], red [T], green [A] and brown [G]. [file 1471-2164-12-116-S4.PDF]

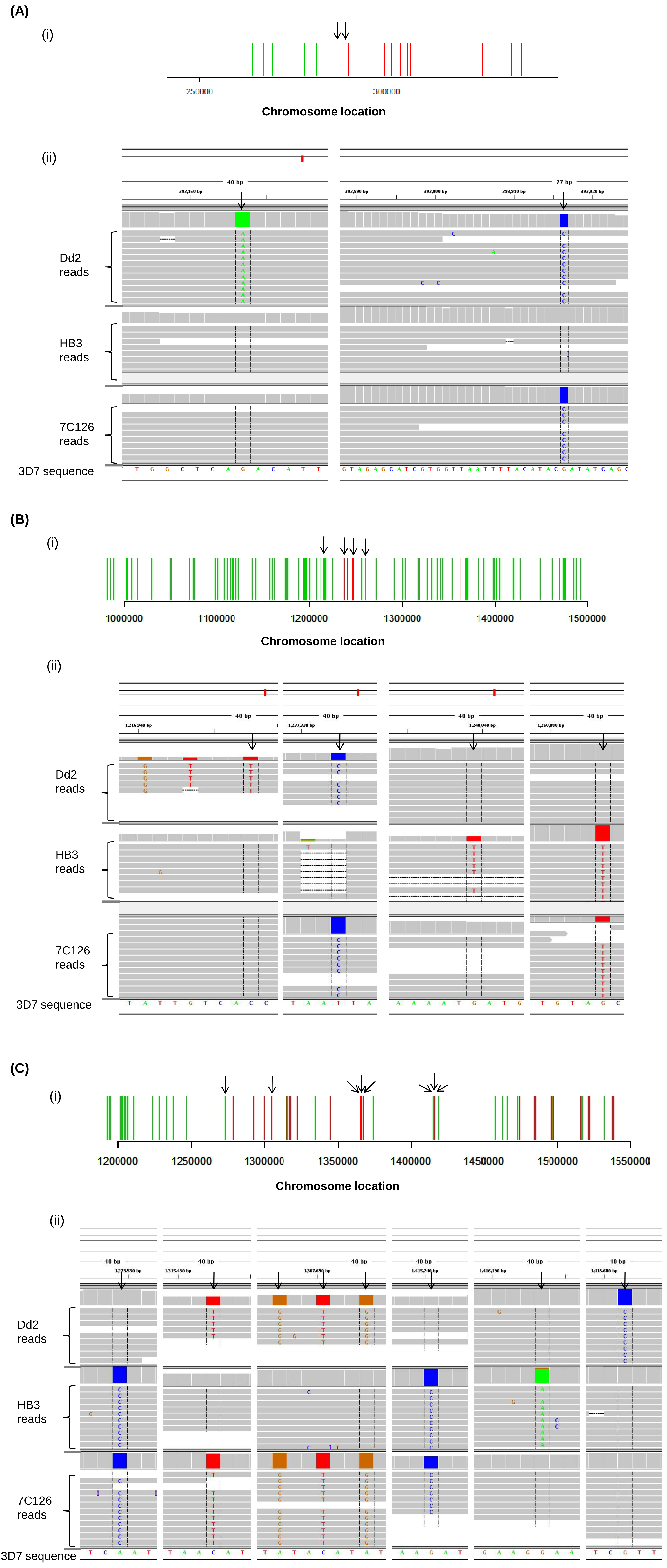

Additional file 4 - Visual inspection of mapped WGS sequence at CO and NCO gene conversion breakpoints shown in Figure 4.

(A) simple CO breakpoint in 7C126 , (B) NCO gene conversion in SC05 and (C) complex CO breakpoint accompanied by a conversion tract (defined by rapid allele changes at/near breakpoint) detected in 7C126. Chromosomal alignments at CO and NCO regions were visually inspected in comparison with the parental genomes, using Integrative Genomics Viewer [45]. (i) SNPs detected by 454 sequencing are ordered according to the chromosomal location (PlasmoDB v5.4 [27]). Each line represents a single SNP marker. HB3 alleles are shown in green bars and Dd2 alleles are shown in red. (ii) Comparison of SNPS and read alignments at selected SNP loci (arrow). SNPs are highlighted in blue [C], red [T], green [A] and brown [G].
