# Supplementary material for: High-throughput 454 resequencing for allele discovery and recombination mapping in Plasmodium falciparum
Source: BMC Genomics. 2011 Feb 17;12:116. doi: 10.1186/1471-2164-12-116 (PMC3055840; doi:10.1186/1471-2164-12-116)
Supplement: Additional file 5 — Resequencing results of de novo SNP positions [file 1471-2164-12-116-S5.PDF]

Additional file 5 - Resequenced *de novo* SNP positions

Chromosome	Reference genome (PlasmoDB 5.4)		Parent genome		Progeny genome		PlasmoDB SNP identity	
	Location	3D7	Dd2	HB3	7C126	SC05	Dd2	HB3
3	207603	C	C	C	C	T	C	C
3	207607	C	C	C	C	T	C	C
6	1068690	T	T	T	T	A	T	T
6	1068694	T	T	T	T	A	T	T
9	275669	C	C	C	T	C	C	C
11	950298	C	C	C	C	T	C	C
11	950301	T	T	T	T	A	T	T
13	983216	C	C	C	A	C	C	C
14	1242888	T	T	T	C	T	T	T
14	2754963	T	T	T	T	A	T	T

Predicted *de novo* SNPs are highlighted in grey
