# Supplementary material for: High-throughput 454 resequencing for allele discovery and recombination mapping in Plasmodium falciparum
Source: BMC Genomics. 2011 Feb 17;12:116. doi: 10.1186/1471-2164-12-116 (PMC3055840; doi:10.1186/1471-2164-12-116)
Supplement: Additional file 6 — Distance between consecutive de novo SNPs. The distance between consecutive de novo SNPs were calculated to detect SNP clustering characteristic of sequencing errors or mis-mapping errors. 35% of the de novo SNPs were clustered in distances of less than 5 bps. [file 1471-2164-12-116-S6.DOC]

**Additional file 6 – Distance between consecutive *de novo* SNPs.**

The distance between consecutive *de novo* SNPs were calculated to detect SNP clustering which is characteristic of sequencing errors or mis-mapping errors. 35% of the *de novo* SNPs were clustered in distances of less than 5 bps.
