# Supplementary material for: High-throughput 454 resequencing for allele discovery and recombination mapping in Plasmodium falciparum
Source: BMC Genomics. 2011 Feb 17;12:116. doi: 10.1186/1471-2164-12-116 (PMC3055840; doi:10.1186/1471-2164-12-116)
Supplement: Additional file 7 — Base conversion trends in de novo SNPs. The type of base conversion was investigated for positions at which the parental base calls were identical. More transversions were detected for SC05 compared to 7C126 (A), but did not show a predominant base conversion bias from 7C126 (B). [file 1471-2164-12-116-S7.DOC]

(A)

(B)

**Additional file 7 – Base conversion trends in *de novo* SNPs.**

The type of base conversion was investigated for positions at which the parental base calls were identical. More transversions were detected for SC05 compared to 7C126 (A), but did not show a predominant base conversion bias from 7C126 (B).
