# Supplementary material for: High-throughput 454 resequencing for allele discovery and recombination mapping in Plasmodium falciparum
Source: BMC Genomics. 2011 Feb 17;12:116. doi: 10.1186/1471-2164-12-116 (PMC3055840; doi:10.1186/1471-2164-12-116)
Supplement: Additional file 8 — Alternate SNP positions. The alternate SNP positions were assessed for their primary and secondary positions base call identity. Most primary base calls reflected the parental base call. The secondary base call position varied in the 2 progeny genomes in base call identity. Majority of the secondary base calls were parental in 7C126, whereas majority of the secondary base calls were non-parental in SC05 (A). Majority of the primary base calls were transitions in 7C126, while they were transversions in SC05 (B, C). [file 1471-2164-12-116-S8.DOC]

**Additional file 8 – Alternate SNP positions.**

The alternate SNP positions were assessed for their primary and secondary positions base call identity. Most primary base calls reflected the parental base call. The secondary base call position varied in the 2 progeny genomes in base call identity. Majority of the secondary base calls were parental in 7C126, whereas majority of the secondary base calls were non-parental in SC05 (A). Majority of the primary base calls were transitions in 7C126, while they were transversions in SC05 (B, C).
