# Supplementary material for: High-throughput 454 resequencing for allele discovery and recombination mapping in Plasmodium falciparum
Source: BMC Genomics. 2011 Feb 17;12:116. doi: 10.1186/1471-2164-12-116 (PMC3055840; doi:10.1186/1471-2164-12-116)
Supplement: Additional file 9 — Selected CNVs in 7C126 and SC05. Comparative genomic hybridization (CGH) was used to detect large (> 1 kb) CNV regions in 7C126 (A) and SC05 (B). Five known CNVs that exist between parental strains were detected in the progeny in Chr 2, 5, 9 and 12. [file 1471-2164-12-116-S9.PDF]

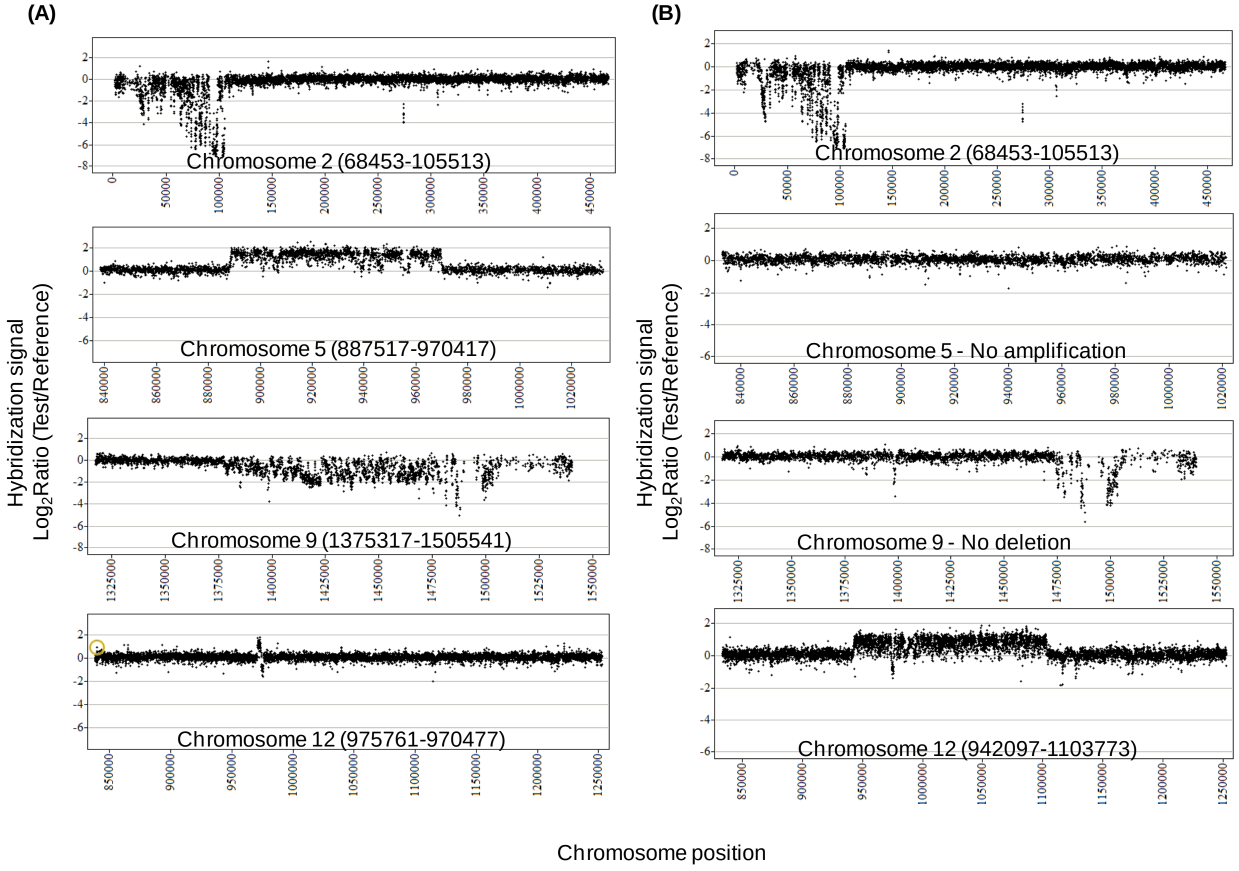

Additional file 9 – Selected CNVs in 7C126 and SC05.

Comparative genomic hybridization (CGH) was used to detect large (> 1 kb) CNV regions in 7C126 (A) and SC05 (B). Five known CNVs that exist between parental strains were detected in the progeny in Chr 2, 5, 9 and 12.
